# Implementation of isoniazid preventive therapy in southern Lima, Peru: an analysis of health center characteristics

**DOI:** 10.1186/s40249-021-00845-0

**Published:** 2021-05-07

**Authors:** Roberto Zegarra-Chapoñan, Lily Victoria Bonadonna, Courtney M. Yuen, Martha Brigida Martina-Chávez, Jhon Zeladita-Huaman

**Affiliations:** 1 Universidad María Auxiliadora, Lima, Peru; 2 Wayne State University School of Medicine, Detroit, MI USA; 3 Harvard Medical School, Boston, MA USA; 4 Universidad Nacional Mayor de San Marcos, Lima, Peru

**Keywords:** Contacts, Chemotherapy, Latent tuberculosis, Children, Adolescent

## Abstract

**Background:**

Tuberculosis (TB) prevention through the use of preventive treatment is a critical activity in the elimination of TB. In multiple settings, limited staffing has been identified as a barrier to managing preventive treatment for TB contacts. This study aims to determine how health center staffing, service type, and TB caseload affects implementation of isoniazid preventive therapy (IPT) for TB contacts in southern Lima.

**Methods:**

We conducted an ecological study in 2019 in southern Lima, Peru. Through the review of medical records, we identified contacts of TB patients who initiated IPT during 2016–2018, and who were 0–19 years old, the age group eligible for IPT according to Peruvian guidelines. We assessed bivariate associations between health center characteristics (numbers of physicians and nurses, types of services available, annual TB caseload) and IPT initiation and completion using binomial logistic regression.

**Results:**

Among 977 contacts, 69% took more than a week to start IPT and 41% did not complete IPT. For those who successfully completed IPT, 58% did not complete full medical follow-up. There was no significant difference in IPT completion or adherence based on whether health centers had more physicians and nurses, more comprehensive services, or higher TB caseloads. Among contacts, female sex was associated with delay in initiating IPT (*P* = 0.005), age 5–19 years old was associated with completion of IPT (*P* = 0.025) and age < 5 years old was associated with completion of clinical evaluations (*P* = 0.041).

**Conclusions:**

There are significant gaps in IPT implementation in health centers of southern Lima, Peru, but insufficient staffing of health centers may not be responsible. Further research is needed to identify how IPT implementation can be improved, potentially through improving staff training or monitoring and supervision.

**Graphic abstract:**

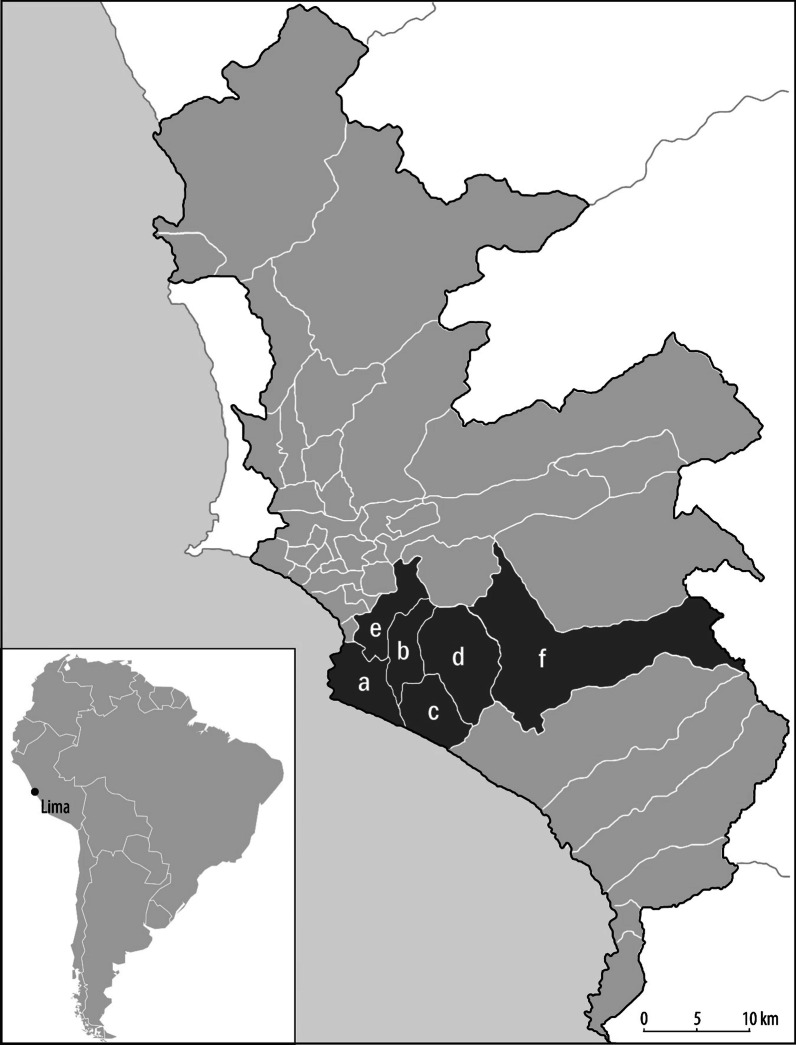

## Background

Tuberculosis (TB) is one of the world's leading causes of mortality, with 10 million people developing the disease in 2019 [1]. In the Americas region, only 65% of suspected TB cases are detected, and pediatric TB accounts for 5.5% of total cases [2]. Peru has the second highest number of TB cases among countries in the Americas region [2]. Given such a high disease prevalence, prevention of TB through the use of preventive treatment is critical for elimination strategies [3]. Contacts of TB patients are a priority group for preventive treatment because of their high risk of developing TB disease [4]. Prompt initiation of preventive treatment, appropriate monitoring, and support for contacts can reduce the risk of developing TB in exposed individuals [5].

Proper implementation of TB preventive treatment for contacts of TB patients could prevent the deaths of thousands of children and adolescents around the globe [6]. However, in many low- and middle-income countries, implementation of preventive treatment for child contacts is poor [7]. Barriers to preventive treatment include lack of awareness among both caregivers and health care workers, as well as logistical challenges in accessing care, such as the cost of transport to attend clinic visits [7, 8].

Appropriate staffing and training of health personnel are important factors in improving TB prevention [9]. Insufficient staffing has been identified as a barrier to TB contact management in multiple studies, since repeated follow-up visits for monitoring contacts on preventive treatment adds substantially to the workload of health care staff who are also monitoring treatment for TB patients [7]. In 1990, Peru’s National TB Program became a national health priority, and since then there has been increased funding and political commitment to TB services, including ensuring adequate staffing [10]. Despite this, Lima continues to face a lack of qualified and well-trained health care staff in the city’s most impoverished areas, where the public health system may lose healthcare staff to private or international health bodies [11]. It is unclear how these staffing challenges affect contact management.

In addition to staffing, other aspects of health centers related to their resources and infrastructure may affect the implementation of preventive treatment for TB contacts. In a study from the Philippines, health centers operated by non-governmental organizations evaluated more child contacts than local government health facilities, in part because they had sufficient resources to conduct home visits [12]. In a study from Brazil, TB contacts who attended family health units, which are designed to provide integrated care for families, were more thoroughly evaluated and more likely to be prescribed isoniazid preventive therapy (IPT) than those who attended basic health units with a more traditional individual care model [13]. In Peru, contradictory results were found by a study that showed that health centers that managed more TB patients were more likely to initiate IPT for contacts < 5 years old but less likely to initiate IPT for contacts 5–19 years old [14].

To improve TB prevention, it is essential to generate evidence about how health center staffing and infrastructure affect the delivery of TB preventive services. The objective of this study was to determine how health center staffing, service type, and TB caseload affects implementation of IPT for TB contacts in southern Lima.

## Methods

### Study design and setting

In 2019, we conducted an ecological study to analyze the relationship between health center staffing, type, and TB caseload, and indicators of IPT implementation in southern Lima, Peru. This study was conducted in the regional public health network of southern Lima, under management of the Lima South Directorate of Integrated Networks (DIN). The Lima South DIN manages 117 health centers that provide TB services, located in 13 city districts, and covering a combined catchment population of 1 860 382 people. Each health center has a TB treatment program with dedicated staff. According to Peruvian guidelines, IPT is indicated for TB contacts who are < 5 years old as well as those who are 5–19 years old and have a positive tuberculin skin test result, after ruling out active TB disease [15]. For IPT, staff dispense isoniazid weekly to caregivers who are responsible for supervising treatment for child and adolescent contacts.

**Fig. 1:**
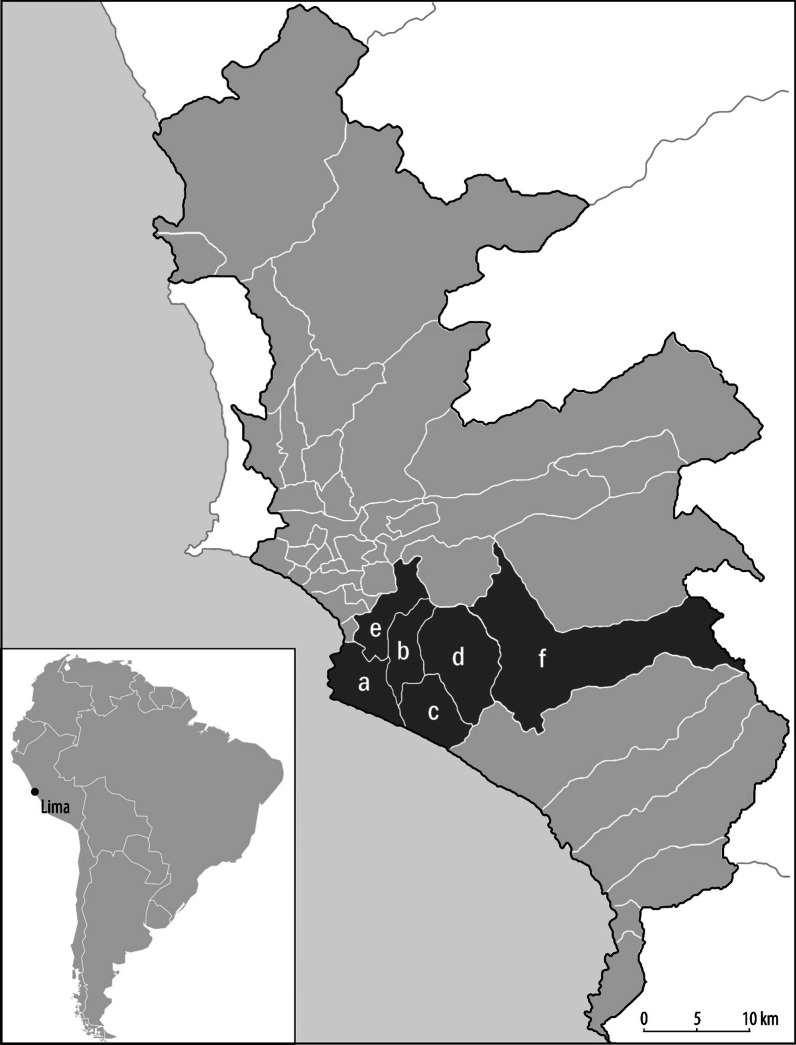
Study districts. Overview of the study area, which covers the followed districts: **a** Chorrillos, 112 contacts identified (11.5%); **b** San Juan de Miraflores, 266 contacts identified (27.2%); **c** Villa El Salvador, 349 contacts identified (35.7%); **d** Villa María del Triunfo, 237 contacts identified (24.3%); **e** Surco, 5 contacts identified (0.5%); **f** Pachacamac, 8 contacts identified (0.8%)

We conducted this study in 46 health centers in the Lima South DIN, located in 6 districts of metropolitan Lima (Fig. 1). We ranked 117 health centers by their TB caseloads recorded in 2016, and identified the top 50 centers with the highest case burdens. Collectively, these health centers treated approximately 80% of diagnosed TB cases in the Lima South DIN [16]. We thus excluded from consideration health centers with very low TB caseloads (generally fewer than 10 patients per year), as our priority was to understand IPT implementation in the health facilities that manage the majority of TB contacts. We approached all 50 health centers and 46 (94%) agreed to participate in the study.

### Data collection

We extracted data from health records of patients who were family members or household contacts of patients receiving treatment for TB between 2016 and 2018, who were 19 years of age or younger and who initiated IPT. We excluded contacts for whom information on age, sex, or IPT completion was missing; who were diagnosed with TB after initiating IPT; and for whom IPT was suspended for other medical reasons.

The outcomes of interest were indicators of the quality of IPT implementation, assessed at the patient level. These included: (1) delayed IPT initiation, defined as > 7 days between initiation of TB treatment in the index case and initiation of IPT in the contact; (2) completion of IPT, defined as the contact taking isoniazid for 24 weeks or 168 doses; and (3) clinical evaluation of contacts, which was considered complete when the contact attended two medical appointments during IPT and one follow-up medical appointment after IPT completion.

Health center-level variables of interest were: (1) number of physicians at the facility; (2) number of nurses at the facility; (3) caseload of TB at the facility reported in 2016; and (4) type of healthcare center. In Peru, three different types of health centers are present in the primary care system: I-2 centers are outpatient health clinics with basic services, I-3 centers are larger health centers without overnight beds, but with some specialist services available, and I-4 centers have overnight beds. In addition, we evaluated associations between age and sex of the contacts and indicators of IPT implementation. We obtained information on physician and nurse staffing from the Lima South DIN administrative office.

### Statistical analysis

To assess the significance of bivariate associations between predictors and outcomes, we generated *P*-values using a binomial logistic regression with robust standard errors to account for clustering at the health center level. A *P*-value < 0.05 was considered statistically significant. Statistical analysis was performed using SAS v9.4 (SAS Institute Inc, Cary, NC, USA).

### Ethical approval

The study was approved by the Institutional Ethics Committees of the Maria Auxiliadora University (No. 005-2019). A waiver of informed consent was granted because data were collected from routine medical records in a way that did not permit the identification of patients.

## Results

We identified 1010 contacts that met the inclusion criteria and obtained information from 977 (99%). Of these, 772 individuals were contacts of index patients with sputum smear-positive TB. The sociodemographic and clinical characteristics of contacts are described in Table 1. Overall, 616 (63%) contacts were under 5 years of age, 551 (56%) were male, and 925 (95%) were contacts of index patients with pulmonary TB.

**Table 1:** Sociodemographic and clinical descriptions of tuberculosis contacts in 46 health facilities of southern Lima, Peru, 2016–2018 (*n* = 977)

Characteristics	Category	*n* (%)
Contact age range	Under 5 years old	616 (63)
	5 to 19 years	361 (37)
Contact sex	Male	551 (56)
	Female	426 (44)
Index patient tuberculosis diagnosis	Pulmonary	925 (95)
	Extrapulmonary	52 (5)
Index patient sputum smear microscopy result	Positive	772 (79)
	Negative	205 (21)
Index patient treatment completion^a^	Successful	833 (85)
	Not successful	135 (14)
	No information	9 (1)

^a^Treatment outcome categories follow standard WHO definitions; successful outcomes include cure and treatment completion, while unsuccessful outcomes include death, loss to follow-up, and treatment failure [17]

Regarding the health center level variables, 41% of health centers had a full-time physician and 13% had 2 or more nurses working in the TB service. As listed in Table 2, 46% of health centers reported serving between 25 and 49 TB patients per year.

**Table 2:** Staffing, tuberculosis caseload, and type of southern Lima health facilities included in the study, 2016 (*n* = 46)

Characteristic	Category	Number of primary health centers, *n* (%)
Physician staffing	1 Full-time	19 (41)
	1 Part-time	27 (59)
Nurse staffing	≥ 2 Full-time	13 (28)
	1 Full-time or Part-time	33 (72)
Number of TB cases per year	≥ 50	10 (22)
	25–49	21 (46)
	11–24	15 (33)
Type	I-2 (Outpatient clinic)	26 (57)
	I-3 (Health center)	9 (20)
	I-4 (Health center with overnight beds)	11 (24)

### Implementation of IPT

**Fig. 2:**
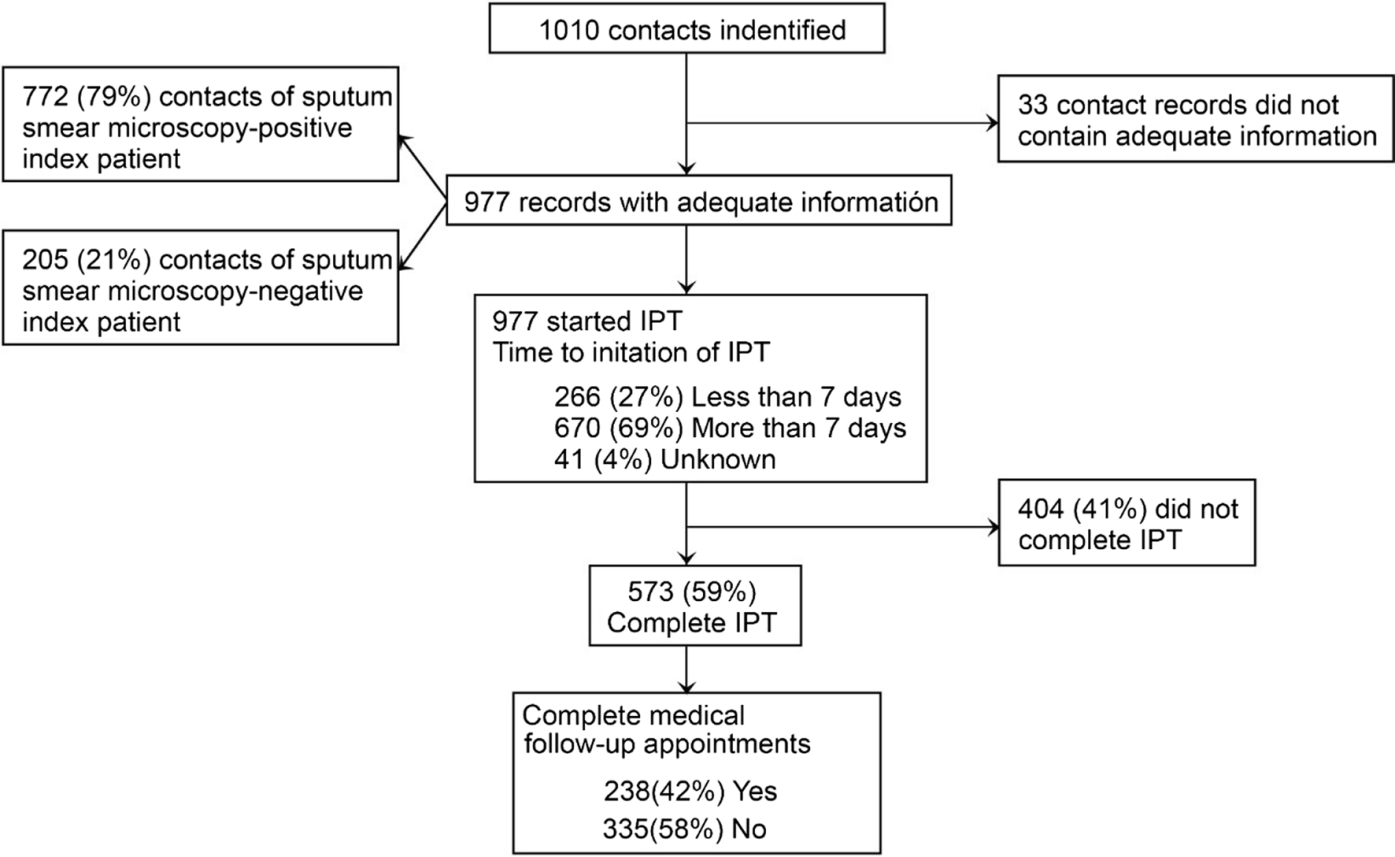
Flow diagram of data collection. IPT: isoniazid preventive therapy

The indicators of IPT implementation are shown in Fig. 2. Of the contacts who began IPT, 670 (72%) did not begin until seven or more days after the index patient began TB treatment and 404 (41%) did not complete treatment. The median number of days of delay initiating IPT was 12 (interquartile range 6–24). Of the contacts who completed IPT, 335 (58%) did not complete the three required medical evaluations.

### Factors associated with IPT implementation

The bivariate analysis (Table 3) reported that there was no significant difference in IPT initiation or completion at health centers with more physicians and nurses. There were also no significant differences in IPT implementation indicators based on health center caseload or type. The percentage of female contacts who delayed IPT initiation (31%) was significantly higher than male contacts (24%) who delayed (*P* = 0.005). IPT completion was significantly greater (*P* = 0.025) in contacts age 5–19 years old (64%) compared to contacts under 5 years of age (56%). In addition, the percentage of contacts age 5–19 years old who completed medical follow-up (46%) was significantly higher (*P* = 0.041) than contacts under 5 years of age (35%).

**Table 3:** Bivariate associations between isoniazid preventive therapy implementation indicators and characteristics of contacts and health centers in southern Lima, 2016–2018

Indicator	Description	Total number	Delayed < 7 days in beginning IPT^a^		Completed IPT		Completed 3 clinical appointments for IPT	
			*n* (%)	*P*-value	*n* (%)	*P*-value	*n* (%^b^)	*P*-value
Age of contact	< 5 years	616	179 (29)	0.110	343 (56)	0.025	157 (46)	0.041
	5–19 years	361	87 (24)		230 (64)		81 (35)	
Sex of contact	Male	551	132 (24)	0.005	320 (58)	0.579	138 (43)	0.378
	Female	426	134 (31)		253 (59)		100 (40)	
Physician staffing	1 full time	581	147 (25)	0.353	346 (60)	0.795	148 (46)	0.331
	1 part time	396	118 (30)		227 (57)		80 (35)	
Nurse staffing	≥ 2 full-time	467	134 (29)	0.607	303 (64)	0.148	144 (48)	0.245
	1 at full-time or part-time	510	510 (26)		270 (53)		93 (35)	
Number of TB cases per year	≥ 50	399	103 (26)	Ref	243 (61)	Ref	102 (42)	Ref
	25–49	374	113 (30)	0.376	215 (57)	0.722	80 (37)	0.712
	11–24	204	50 (25)	0.663	115 (56)	0.687	46 (49)	0.640
Health center type	I-2	401	98 (24)	Ref	245 (61)	Ref	108 (44)	Ref
	I-3	207	64 (31)	0.435	107 (52)	0.452	34 (32)	0.338
	I-4	369	104 (28)	0.492	221 (59)	0.900	96 (43)	0.962

*IPT* isoniazid preventive therapy, *TB* tuberculosis

^a^Excludes 41 contacts with insufficient data to determine days of delay to initiate IPT

^b^Of those who completed IPT

## Discussion

We found significant gaps in IPT implementation in health centers of southern Lima, Peru, but insufficient staffing of health centers may not be responsible. Among TB contacts 0–19 years old who initiated IPT, 72% initiated over a week after the index patient was diagnosed with TB and 41% did not complete IPT treatment. Studies from diverse settings have qualitatively identified insufficient staffing as a barrier to IPT implementation for child contacts [18–20], and we sought to evaluate this issue quantitatively. In our setting, we found no association between health center staffing, type, or TB caseload and IPT initiation delay, IPT completion, or completion of clinical follow-up.

One explanation for our results may be that IPT management is more affected by healthcare staff training, organization, and monitoring rather than staff quantity. Other studies have shown substantial knowledge gaps exist among health care workers responsible for IPT management in high-burden settings [19, 20]. Providing health care workers with training and simple tools for IPT management has also been shown to improve performance [21]. Additionally, the main drivers of suboptimal IPT completion may be factors outside the health system. For example, lack of resources for transport to clinic visits [22] and caregivers’ perception that treatment is not important for healthy children [22, 23] have been identified as barriers to IPT completion in other high-burden settings.

Despite our results, staffing and other aspects of health center capacity may still be important for contact management. This is especially important when considering earlier points in the TB contact management care cascade. Our study only collected data for children who initiated IPT, and so we were unable to evaluate the impact of health center staffing and capacity on contact identification and evaluation. This process may be particularly reliant on staffing, as the number of TB contacts involved is often large and household visits are important for ensuring medical evaluation [12, 20]. Other studies have shown the greatest gaps in contact management to occur during this identification and screening process [24, 25]. Completing medical evaluation also requires radiographic imaging and TB testing availability. Smaller outpatient clinics that lack this capacity may face challenges in initiating children on IPT [26, 27].

We found that male contacts initiated IPT earlier than female contacts and contacts 5–19 years old were more likely to complete treatment and medical evaluation compared to contacts < 5 years old. Female contacts may delay more because of gender and social norms in Peru that prioritize men’s health over women in general. Other studies have found this to be true, specifically in the context of TB treatment [28, 29]. The higher treatment completion among older contacts may be due to the difficulty of administering pills to young children [30, 31], as pediatric formulations of isoniazid are not available in Peru. Greater autonomy of adolescent contacts and less reliance on caregivers may have also contributed to this result. Since our study did not primarily focus on individual-level predictors of IPT completion, these preliminary findings could guide future research in understanding and addressing disparities in IPT service delivery.

This study includes several limitations. First, we only collected data on contacts who initiated IPT and so we could not evaluate the percentage of contacts who were eligible for but did not receive IPT. This subset of contacts may be more affected by health center resources and staffing. Future research might evaluate the association between health center characteristics and poor prescription of IPT—a recognized gap in TB contact care in Peru [32]. Secondly, we only included the highest TB burden health centers in our study and therefore, our results may not apply to health centers with fewer TB cases. Finally, we collected limited socio-demographic data on index patients and contacts. Thus, we were unable to complete a multivariable analysis that may have allowed us to better understand the sex and age disparities we observed. This is an important focus of further research.

## Conclusions

In an effort to quantitatively measure a barrier to IPT identified by previous qualitative research, we found that health center staffing and capacity were not associated with key IPT indicators in Lima, Peru. This does not, however, mean that these factors are unimportant. Indeed, we found that substantial proportions of child contacts were delayed in initiating IPT and that completion of both IPT and clinical follow-up was suboptimal. Improvements in the health system will be important for addressing these gaps in the care cascade. Our study suggests that the solution will not be as simple as providing more staff. Further research to evaluate the quality of care being delivered as well as patient-related barriers is necessary to identify the interventions needed to improve management of TB contacts.

## Data Availability

The datasets analyzed during the current study are available from the corresponding author on reasonable request.

## Abbreviations

TB: Tuberculosis
IPT: Isoniazid preventive therapy
DIN: Directorate of Integrated Networks
